# Not All Frailty Assessments Are Created Equal: Comparability of Electronic Health Data-Based Frailty Assessments in Assessing Older People in Residential Care

**DOI:** 10.1177/10998004241254459

**Published:** 2024-05-13

**Authors:** Jonathan Kong, Kelly Trinh, Kathrine Hammill, Carla Chia-Ming Chen

**Affiliations:** 1 8001 James Cook University, Douglas, QLD, Australia; 2 Helping Hand Aged Care, Tranmere, SA, Australia; 3 170512 Data61, CSIRO, Research Way, Clayton, VIC, Australia; 4 Enable Me Occupational Therapy, Moorebank, NSW, Australia

**Keywords:** frailty, aged care, assessment, electronic health records

## Abstract

**Objectives:** To evaluate the comparability of frailty assessment tools – the electronic frailty index (eFI), retrospective electronic frailty index (reFI), and clinical frailty scale (CFS) – in older residents of care facilities. **Methods:** Data from 813 individuals aged 65 or older, with frailty and co-morbidities, collected between 2022 and 2023, were analysed using various statistical methods. **Results:** The results showed significant differences in frailty classification among the tools: 78.3% were identified as moderately to severely frail by eFI, 59.6% by reFI, and 92.1% by CFS. Statistical tests confirmed significant differences (*p* < .05) in their assessments, indicating variability in measurement methods. **Discussion:** This study advances the understanding of frailty assessment within aged-care settings, highlighting the differences in the efficacy of these assessment tools. It underscores the challenges in frailty assessments and emphasizes the need for continuous refinement of assessment methods to address the diverse facets of frailty in aged care.

## Introduction

Frailty is prevalent in older people, marked by diminished strength and endurance, heightened susceptibility to stressors, and delayed recovery from illness or injury (Chen et al., 2014; Clegg et al., 2013; Fried et al., 2001; Rockwood & Howlett, 2018; Wleklik et al., 2020). It presents risks such as a reduced quality of life, increased healthcare costs, and an increased mortality risk (Hoogendijk et al., 2019; Ofori-Asenso et al., 2019). To effectively address these risks, accurate, reliable, and reproducible frailty assessments are necessary across diverse settings.

The process of selecting an appropriate frailty measurement instrument is inherently complex, as it involves navigating a landscape populated with a multitude of assessments, each characterized by unique methodologies and scoring systems. This diverse array is exemplified by instruments such as Fried Frailty Phenotype (FFP), Frailty Index (FI), Groningen Frailty Indicator (GFI), Edmonton Frail Scale (EFS), PRISMA-7, SHARE-FI, and Tilburg Frailty Indicator (TFI), to name but a few (Bielderman et al., 2013; Clegg et al., 2016; Drubbel et al., 2013; Faller et al., 2019; Fried et al., 2001; Gobbens & Uchmanowicz, 2021; Hoffmann et al., 2020; Kim, 2020; Kim et al., 2018; NSW Agency for Clinical Innovation, 2019; Oviedo-Briones et al., 2021; Perna et al., 2017; Peters et al., 2012; Raîche et al., 2008; Rockwood et al., 2005; Rockwood & Theou, 2020; Rolfson et al., 2006; Romero-Ortuno, 2011; Romero-Ortuno et al., 2010). It is important to acknowledge that these assessments, which vary from self-administered questionnaires to combinations of observations and self-reports, do not conform to a singular, standardized approach. Currently, there is no universally accepted definition of frailty, nor a standardized system for its assessment, leading to variations in understanding and approach in the international community (Abbasi et al., 2019; Broad et al., 2020; Brundle et al., 2019; Hoogendijk et al., 2019; Oliosi et al., 2022; Oviedo-Briones et al., 2021). Additionally, most existing frailty instruments focus on community-dwelling individuals, leaving a gap in understanding frailty assessment for older people in residential care facilities (Buckinx et al., 2017; Ga et al., 2018).

In the context of assessing frailty within populations characterized by severe levels of this condition, particularly those residing in residential care facilities, there is a need for specialized assessment tools. This necessity arises from the unique challenges inherent to these settings. Many of the contemporary frailty assessment tools, as mentioned earlier, may present implementation difficulties in such settings. This is primarily because these instruments were initially developed for use with elderly who are typically more ambulatory and have better cognitive function. Consequently, they often require participants to have the ability to comprehend and adhere to instructions, a capability that might not be present in all individuals within severely frail populations (Bieniek et al., 2016; Buckinx et al., 2017). Individuals in residential care facilities may struggle with these assessments due to cognitive impairments, limited mobility, and difficulty comprehending the tasks. Therefore, it is necessary to explore how frailty is assessed in this population and enhance comparability among existing frailty tools. The development and application of frailty assessments specifically tailored for aged-care settings have begun to address this gap. Particularly among these are the FRAIL-NH scale (Ge et al., 2019; Liau et al., 2021; Theou et al., 2016) and the retrospective electronic Frailty Index (reFI) (Sarwar et al., 2022). These instruments significantly expand the scope of frailty assessments to encompass individuals residing in aged-care settings, thus facilitating a more comprehensive and inclusive understanding of frailty across diverse residential contexts. The development of these tools reflects a growing awareness of the unique care requirements of older adults in care facilities and highlights the significance of adopting customized approaches in both the assessment and management of frailty. Nonetheless, this field of study warrants further research to enhance the effectiveness and applicability of these tools across different aged-care scenarios.

In recent years, the Australian Government rolled out the Australian National Aged Care Classification (AN-ACC) framework, marking a significant advancement in the nation's aged care strategy. Developed from key studies (Eagar et al., 2019, 2020), AN-ACC serves both as a funding mechanism and as an assessment tool, evaluating aspects such as mobility, functional abilities, daily living task performance, pressure sore risk, behavior, and fall risks. This framework is specifically designed to better gauge the funding needs of frail older people in aged care homes. It categorizes funding into 13 distinct levels that reflect the diverse care requirements of residents, from minimal to highly complex. Assessments are conducted on-site by independent teams, ensuring objective and standardized evaluations that closely align financial support with actual care needs. This approach aims to enhance fairness and transparency in resource distribution across various facilities and regions, promoting efficiency within the sector. Despite its innovative design, there remains a notable gap in research, particularly in how existing frailty assessments align with AN-ACC categories. This alignment is critical as accurate frailty assessments are essential for determining appropriate care levels and funding. Further research is necessary to evaluate the framework's effectiveness and guide future improvements and policy decisions in aged care services. The knowledge from such research could also offer insights into aged care systems internationally, enhancing global approaches to similar challenges.

This study addressed two primary research questions. Firstly, we examined the comparability of frailty assessment tools (eFI, reFI and CFS) in residential care facilities to understand how these tools align in assessing frailty within this context. Secondly, we examined how frailty scores from these tools impact AN-ACC care classification, exploring their influence on AN-ACC classification.

## Methods

### Study Design and Population

In this retrospective cross-sectional study, we utilized de-identified electronic health records obtained from ten residential aged-care facilities with the support of an aged care organization in South Australia. This study was approved by the Human Research Ethics Committee of James Cook University (Ethics Approval Number H9171). Due to the secondary nature of the study, the Human Research Ethics Committee of James Cook University has granted a waiver for obtaining consent, and approval for the use of data was obtained from the aged-care organization. The study exclusively relied on anonymized data from existing aged-care databases and routinely collected health and clinical data, eliminating the need for direct patient contact or data acquisition outside of these databases.

The inclusion criteria for this study comprised individuals aged 65 and older residing in residential care facilities between 2022 and 2023. Individuals were required to have completed the AN-ACC assessment, received a CFS score from the clinical nursing team, and have complete electronic health records for reFI and eFI scoring. Those without an AN-ACC assessment or with incomplete health data were excluded from the analysis.

### Outcome Measures for Frailty

In this study, the rationale for selecting the electronic Frailty Index (eFI) (Clegg et al., 2016), the retrospective electronic Frailty Index (reFI) (Sarwar et al., 2022), and the Clinical Frailty Scale (CFS) (Rockwood et al., 2005; Rockwood & Theou, 2020) is grounded in their distinctive characteristics and compatibility with aged-care settings. Both the eFI and reFI, drawing on a cumulative deficit approach, allow for electronic calculation of frailty, streamlining the assessment process by eliminating manual scoring. This feature is particularly advantageous in aged-care contexts, where efficiency and accuracy are paramount. The eFI, as developed by Clegg and colleagues (Clegg et al., 2016), encompasses a broader spectrum of 36 deficit variables, offering a comprehensive assessment. Conversely, the reFI, introduced by Sarwar (Sarwar et al., 2022), utilizes a more focused set of 32 variables, thus differing in the scope of frailty measurement. The choice of the CFS (Rockwood et al., 2005, 2007; Rockwood & Theou, 2020), based on clinical judgment and requiring assessment by qualified clinical nursing staff, provides a contrast to the data-driven approaches of eFI and reFI. The CFS is straightforward in its application, assessing health domains such as comorbidity, function, and cognition, and categorizing frailty on a scale from 1 to 9. This traditional approach complements the electronic methods, offering a different perspective on frailty assessment.

For this study, the eFI and reFI were developed using routinely collected, de-identified administrative aged care data extracted from the electronic health records system. These data originating from medical history and AN-ACC assessments. To determine matching features, we assessed whether the identified deficits aligned with those defined by the eFI and reFI frailty instruments. We assigned binary values (1 if present, zero if absent) based on the presence of the deficit in the medical history or AN-ACC assessments. This process was replicated for constructing the reFI. Given that all participants in residential care facilities were inherently frail and required significant care, we uniformly applied three variables—housebound status, care requirements, and social vulnerability—to all residents in residential care facilities. For more general deficits, such as activity limitation, mobility and transfer problems, memory, and cognitive issues, we utilized AN-ACC assessments like Resource Utilization Groups – Activities of Daily Living Instrument (RUG-ADL), De Morton Mobility Index (DEMMI), and Australian Functional Measure (AFM). Scoring was determined based on predefined cut-off points (RUG-ADL ≥17, DEMMI ˃ 3, AFM ≤21), indicating the presence of specific deficits if scores were above or below the cut-off (Westera et al., 2019). In the case of CFS, scores assigned by clinical nursing staff were directly extracted from the data. Refer to Table 1 for details on the frailty deficits included in these instruments. The final step involved uploading the data to R Studio to generate eFI and reFI scores. Following the outlined steps, we successfully obtained the eFI, reFI, and CFS scores for 813 participants along with their respective variables for subsequent analysis.

**Table 1.** List of Frailty Deficits Contained in eFI and reFI.

Frailty Deficits contained in eFI	Item	Data Source	Frailty Deficits contained in reFI	Item	Data Source
Activity limitation	1	RUG-ADL assessment	Anemia and hematinic deficiency	1	Medical history
Anemia and hematinic deficiency	2	Medical history	Anxiety	2	Medical history
Arthritis	3	Medical history	Arthritis	3	Medical history
Atrial fibrillation	4	Medical history	Cancer	4	Medical history
Cerebrovascular disease	5	Medical history	Cardiac	5	Medical history
Chronic kidney disease	6	Medical history	Cognition	6	AFM assessment/Medical history
Diabetes	7	Medical history	Dementia	7	Medical history
Dizziness	8	Medical history	Depression	8	Medical history
Dyspnea	9	Medical history	Diabetes	9	Medical history
Falls	10	Falls review/Medical history	Dizziness	10	Medical history
Foot problems	11	Medical history	Dressing, Personal hygiene, and toileting	11	RUG-ADL assessment
Fragility fracture	12	Medical history	Dysphagia	12	Medical history
Hearing impairment	13	Medical history	Dyspnea	13	Medical history
Heart failure	14	Medical history	Fall incidents	14	Falls review/Medical history
Heart valve disease	15	Medical history	Feet or foot problems	15	Medical history
Housebound	16	Applied to residents in care	Fracture	16	Medical history
Hypertension	17	Medical history	Hearing	17	Medical history
Hypotension/syncope	18	Medical history	Hypertension or hypotension	18	Medical history
Ischemic heart disease	19	Medical history	Incontinence (Fecal)	19	Medical history
Memory and cognitive problems	20	AFM assessment/Medical history	Insomnia	20	Medical history
Mobility and transfer problems	21	DEMMI assessment	Mobility	21	DEMMI assessment
Osteoporosis	22	Medical history	Neurological	22	Medical history
Parkinsonism and tremor	23	Medical history	Osteoporosis	23	Medical history
Peptic ulcer	24	Medical history	Pain	24	Medical history
Peripheral vascular disease	25	Medical history	Peripheral vascular disease	25	Medical history
Polypharmacy (meds ≥9)	26	Medications	Polypharmacy (meds ≥9)	26	Medications
Requirement for care	27	Applied to residents in care	Renal	27	Medical history
Respiratory disease	28	Medical history	Respiratory	28	Medical history
Skin ulcer	29	Medical history	Thyroid	29	Medical history
Sleep disturbance	30	Medical history	Ulcers	30	Medical history
Social vulnerability	31	Applied to residents in care	Vision	31	Medical history
Thyroid disease	32	Medical history	Weight loss	32	Weight review/Medical history
Urinary incontinence	33	Medical history			
Urinary system disease	34	Medical history			
Visual impairment	35	Medical history			
Weight loss and anorexia	36	Weight review/Medical history			

### Statistical Analysis

To facilitate the comparability of frailty data and streamline the analysis and interpretation across three different frailty instruments with varied measurement scales, the data were dichotomized into 'Fit-Mildly Frail' and 'Moderate-Severely Frail' categories. This dichotomization was based on the cut-off points outlined in Table 2, which presents the specific thresholds for each category derived from multiple sources (Clegg et al., 2016; Rockwood et al., 2005; Rockwood & Theou, 2020; Sarwar et al., 2022). This approach was employed to simplify the analytical process and enhance the interpretability of the data. Population demographics and frailty distribution were described using descriptive statistics. The baseline characteristics of the residents were compared based on the frailty score for each frailty assessment instrument, using the *t* test for continuous variables and the chi-square test for categorical variables. The statistical agreement between eFI, reFI, and CFS was assessed using the Pearson correlation coefficient, while McNemar and Cohen’s Kappa statistical tests were applied to detect differences in frailty classification across the assessments. Utilising Principal Component Analysis (PCA) as a clustering method, we aimed to identify patterns in deficits associated with frailty, specifically among individuals assessed as fit-mildly frail and moderate-severely frail by eFI, reFI, and CFS. In a comprehensive analysis to elucidate the impact of eFI, reFI, and CFS on the Aged Care Funding Instrument (AN-ACC), we examined the distribution of frailty scores derived from the three assessment tools. All analyses were conducted using R (version 4.2.3) and RStudio (version 2023.06.1 Build 524).

**Table 2.** Operational definition of Frailty Outlined by the Frailty Measurement Instruments.

	Fit-Mildly Frail					Moderate-Severely Frail			
CFS (Rockwood et al., 2005; Rockwood & Theou, 2020)	1: Very fit	2: Well	3: Managing well	4: Vulnerable	5: Mildly Frail	6: Moderately Frail	7: Severely Frail	8: Very severely Frail	9: Terminally Ill
CFS (ver. 2.0) (Rockwood et al., 2005; Rockwood & Theou, 2020)	1: Very fit	2: Fit	3: Managing well	4: Living with very mild frailty	5: Living with mild frailty	6: Living with moderate frailty	7: Living with severe frailty	8: Living with very severe frailty	9: Terminally Ill
eFI (Clegg et al., 2016) (36 items)	0 to 0.12: Fit				>0.12 to 0.24: Mildly Frail	>0.24 to 0.36: Moderately Frail	>0.36: Severely Frail		
reFI (Sarwar et al., 2022) (32 items)	0 to 0.13: Fit				>0.13 to 0.26: Mildly Frail	>0.26 to 0.39: Moderately Frail	>0.39: Severely Frail		

## Results

### Baseline Characteristics

Of the 813 residents, 31.6% were males and 68.4% were females. Female median age was 88, ranging from 67 to 104, whereas the median age of male residents was 85, ranging from 66 to 100. Based on the distribution of the frailty scores of the study population, we classified the residents into “Fit-Mildly Fail” and “Moderately-Severely Frail” based on the cut-off scores outlined in Table 2. As shown in Appendix 1, there was a notable difference in age and in most frailty deficits between the “Fit-Mildly Frail” and “Moderately-Severely” Frail groups, when measured using eFI and reFI instruments. However, the CFS assessment showed no significant differences between these groups, apart from specific conditions such as mobility impairment, depression, dementia, and incontinence, which were found to be more prevalent in the “Moderately-Severely Frail” group. The primary frailty deficit observed among residents was mobility impairments (*n* = 780, 96%), markedly influencing their ability to move independently or without assistance. Following closely, the second most prevalent frailty deficit was hypertension (*n* = 572, 70%), followed by arthritis (*n* = 523, 64%). Residents also commonly experienced depression (*n* = 498, 61%), dementia (*n* = 439, 54%), and pain (*n* = 403, 50%). Additional details are available in Appendix 1.

### Comparability Between CFS, eFI, and reFI

**Figure 1.**
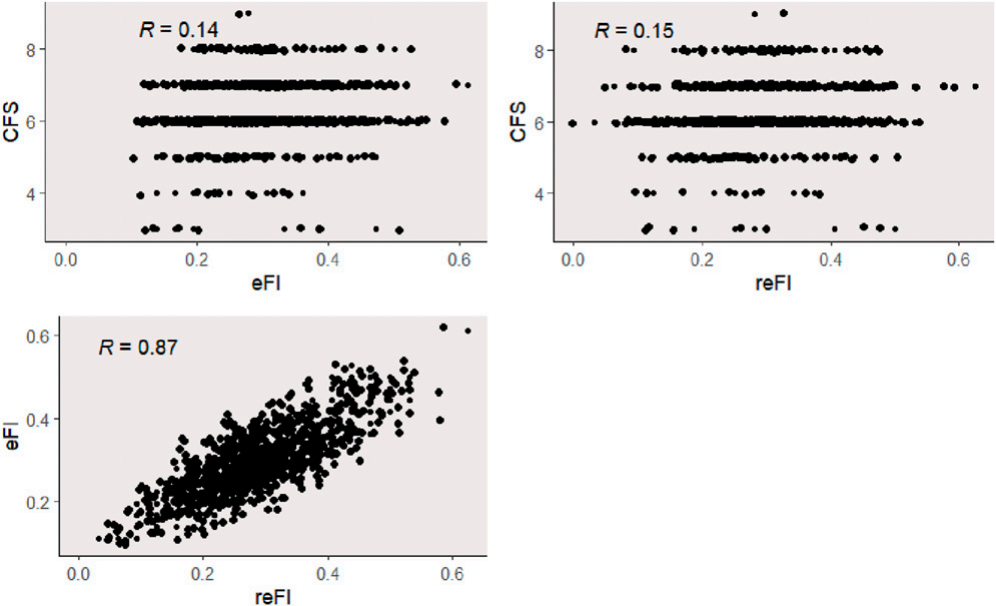
Scatter plots show correlation between (1) eFI and CFS, (2) reFI and eFI, and (3) reFI and CFS). There was a strong correlation between reFI and eFI (Pearson correlation of 0.87).

The comparative analysis between eFI and CFS (Pearson correlation of 0.14), and reFI and CFS (Pearson correlation of 0.15) (Figure 1) revealed no association. There is however a correlation between eFI and reFI (Pearson correlation of 0.87).

As shown in Table 3, upon dichotomizing the data into fit-mildly frail and moderate-severely frail groups, notable differences emerged, particularly between CFS and eFI assessments (McNemar's χ^2^ = 67.44, *p*-value<.05). CFS identified 92.1% (*n* = 749) of residents as moderate-severely frail, while eFI classified only 78.3% (*n* = 637) in the same category. Interestingly, 57.8% (*n* = 37) of the 64 residents labelled fit-mildly frail by CFS were identified as moderate-severely frail by eFI. Conversely, of the 749 residents classified as moderate-severely frail by CFS, 19.8% (*n* = 149) were labelled as fit-mildly frail by eFI. A significant difference also surfaced when comparing CFS and reFI assessments in terms of categorizing individuals into fit-mildly frail and moderate-severely frail groups (McNemar's χ^2^ = 221.96, *p*-value<.05). While 92.1% (*n* = 749) of residents were deemed moderate-severely frail by CFS, only 59.6% (*n* = 485) fell into the same category with reFI. Particularly, 39% (*n* = 25) of the 64 residents classified as fit-mildly frail by CFS were identified as moderate-severely frail by reFI. Similarly, of the 749 residents categorized as moderate-severely frail by CFS, 38.5% (*n* = 289) were labelled fit-mildly frail by reFI. A difference also identified between eFI and reFI assessments (McNemar's χ^2^ = 135.9, *p*-value<.05), approximately half of the residents (*n* = 161, 49%) identified as fit-mildly frail by reFI were classified as moderate-severely frail by eFI. This was consistent with the results from Cohen's Kappa, which indicated little to no agreement between eFI and CFS (kappa = 0.12), reFI and CFS (kappa = 0.08), and eFI and reFI (kappa = 0.53), based on the interpretation of the kappa statistic (McHugh, 2012).

**Table 3.** Comparison of Binary Frailty Classification by eFI, reFI, and CFS.

eFI	CFS	
	Fit – Mildly Frail	Moderate – Severely Frail
Fit – Mildly Frail	27	149
Moderately – Severely Frail	37	600
reFI	CFS	
	Fit – Mildly Frail	Moderate – Severely Frail
Fit – Mildly Frail	39	289
Moderately – Severely Frail	25	460
eFI	reFI	
	Fit – Mildly Frail	Moderate – Severely Frail
Fit – Mildly Frail	167	9
Moderately – Severely Frail	161	476

### Frailty Groupings and Correlations

The Principal Component Analysis (PCA) identified frailty deficits, including peripheral vascular disease, chronic kidney diseases, diabetes, respiratory diseases, dyspnea, urinary diseases, foot problems, polypharmacy, and dizziness, are common in the moderate-severely frail group for both eFI and reFI (see Appendix 2 - Supplementary Figures 1 and 2). Distinctions emerged in the clustering analysis, revealing that the eFI identified moderate-severely frail group had a higher prevalence of heart-related diseases such as heart failure, ischemic heart disease, heart valve diseases, and atrial fibrillation. Interestingly, PCA showed that the deficits mentioned in the moderate-severely frail group in both eFI and reFI were present in both CFS-identified groups (i.e., Fit-Mildly Frail and Moderate-Severely Frail; see Appendix 2 - Supplementary Figures 3 and 4). This suggests that CFS does not deem these deficits crucial for classifying an individual’s frailty.

Moreover, the PCA results demonstrated a high correlation between certain frailty deficits in eFI and reFI among older people in aged-care facilities. For instance, individuals with atrial fibrillation, based on the deficits specified in the reFI assessment, were found to have at least one of the following: foot problems, diabetes, chronic kidney disease, respiratory system disease, skin ulcer, heart failure, and polypharmacy. Similar patterns were observed in the deficits outlined in the eFI assessment (See Appendix 2 - Supplementary Figures 1 and 2).

### Frailty Indices and AN-ACC Classifications

**Figure 2.**
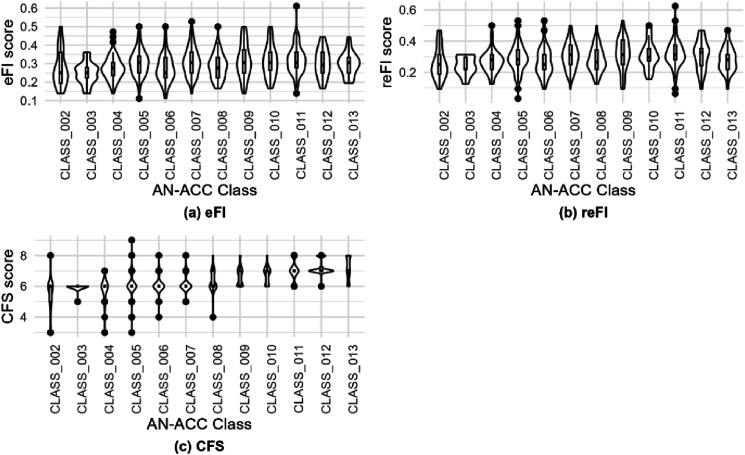
The association between frailty score and AN-ACC classification. (a) Distribution of eFI scores versus AN-ACC, (b) distribution of reFI scores versus AN-ACC, (c) distribution CFS scores versus AN-ACC.

Figure 2 shows the distributions of frailty scores for each AN-ACC classification, considering eFI, reFI, and CFS assessments. eFI frailty scores predominantly fell between 0.2 and 0.4 across all AN-ACC classes. No significant association was observed between eFI frailty scores and AN-ACC classes. Similarly, reFI frailty scores showed no significant association with AN-ACC classes. For CFS frailty scores, considerable variation was noted in individuals with AN-ACC classification 8 and below. Individuals with AN-ACC classification 9 and higher consistently exhibited CFS scores higher than 6. The median CFS score for AN-ACC classification 8 or less was 6, contrasting with a median CFS frailty score of 7 for AN-ACC classifications 9 and above.

## Discussion

In this study, we evaluated the comparability of three frailty assessment tools (eFI, reFI, CFS) in residential care facilities. Additionally, we investigated the impact of frailty scores, as determined by these tools, on the AN-ACC care classification framework. This investigation was conducted in alignment with the research questions we previously outlined, aiming to understand how these tools perform in a residential care context and their influence on care classification outcomes.

In our study, we observed variations in the results produced by these assessment tools, which might suggest differences in their measurement approaches. Specifically, the data did not consistently demonstrate convergent validity between the CFS and the eFI, or between the CFS and the reFI. Our findings align with the study conducted by Broad and colleagues, which similarly reported no convergent validity between CFS and eFI in elderly residents within the UK community (Broad et al., 2020). A comparison with Broad's study disclosed similarities in age distribution but highlighted differences in the proportion of moderate-severely frail participants. In their study, approximately 78% of the participants were 80 years and older, which is comparable to the age distribution of our study (81.35%). However, we had a much higher proportion of CFS defined moderate-severely frail participants in our study. Conversely, Brundle and colleagues found convergent validity between CFS and eFI in a community-based cohort study, attributing variations to differences in study cohorts, including exclusion criteria and age distribution (Brundle et al., 2019). CFS relies on clinical judgment, while eFI and reFI use the cumulative deficit approach, leading to differing outcomes when applied to the same individuals due to inherent variations in their theoretical frameworks and assessment criteria. Another explanation is that frailty index measures may not fully capture frailty levels in those aged over 85, as noted by Swart and colleagues (Swart et al., 2023). The eFI and reFI often assign higher frailty scores to individuals with multiple medical conditions, potentially leading to misclassification of genuine frailty. Moreover, our PCA analysis revealed correlations between deficits in eFI and reFI among elderly people in aged-care facilities, contributing to higher frailty scores for those with comorbidities. For example, the association between atrial fibrillation and various issues, such as foot problems, diabetes, chronic kidney disease, respiratory system disease, skin ulcers, heart failure, and polypharmacy. In contrast, the CFS utilizes a broader range of assessment criteria, aiming for a balanced evaluation that seeks to avoid favoring individuals with multiple medical conditions.

Pijpers and colleagues noting that an increase in deficits or comorbidities does not necessarily lead to a loss of independence with effective management, highlighting the importance of considering both quantity and impact of health conditions for an accurate frailty assessment (Pijpers et al., 2012). Contrary to Broad et al.'s findings of higher eFI scores in the community, our study in residential care facilities showed higher CFS scores compared with eFI or reFI scoring (Broad et al., 2020). This difference may be attributed to our cohort's distinctive characteristics, residing in aged-care facilities, already demonstrating significant frailty compared to Broad's community-dwelling older individuals. The evaluation criteria of CFS, capturing factors particularly relevant to frailer and older individuals, could contribute to this observed distinction. Our study also revealed subtle yet noteworthy variations in the results obtained from eFI and reFI assessments. Despite their methodological similarities in evaluating frailty, these indices yielded slightly different outcomes. This variation may be primarily attributable to differences in the scope and depth of the frailty measurement criteria employed by each index. However, it is important to acknowledge and consider these observations as preliminary, highlighting an area for further research to better understand the distinctions and implications of these variations in frailty assessment.

In our analysis, it appeared that eFI and reFI might not have a significant association with the AN-ACC classifications. This observation might suggest that these electronic assessment tools may not entirely align with the parameters of this specific care classification system. On the other hand, the data seemed to show a slight correlation with CFS, which is more reliant on clinical judgment. This possible correlation could indicate that the CFS may align somewhat more closely with the criteria used in the AN-ACC framework. However, it is important to approach these findings with caution, as they are indicative rather than conclusive. These preliminary observations highlight possible variations in how these frailty assessment tools conceptualize and measure frailty, particularly in relation to the care needs as defined within the AN-ACC system. Such insights call for further investigation to understand more deeply the relationship between frailty assessments and care classification in aged-care settings.

This study has highlighted some key insights into the assessment of frailty in aged-care settings, particularly in the context of the eFI, reFI, and CFS tools. Consistent with existing literature, our findings underscore the inherent complexity of frailty as a multifaceted and multidimensional condition. The diversity of factors influencing the progression of frailty further reinforces the notion that a singular assessment tool may not suffice for a comprehensive evaluation. Consequently, this suggests the potential benefit of employing a combination of assessment tools or exploring multi-dimensional assessment strategies to better capture the complexities of frailty.

Additionally, the methodological approach of dichotomizing data into “Fit-Mildly Fail” and “Moderately-Severely Frail” categories warrants reconsideration. It is important to acknowledge, however, that this method of categorization, while beneficial in reducing complexity and enhancing clarity, inherently involves a degree of simplification. Consequently, it may not fully encapsulate the progression of frailty that exists on a continuum. As such, the results derived from this dichotomized data should be interpreted with a degree of caution, recognizing the potential limitations inherent in this approach. This dichotomization was intended not as a definitive representation of the frailty spectrum, but rather as a pragmatic tool to aid in the comparative analysis, bearing in mind the inherent trade-offs between analytical simplicity and the richness of detailed data. Future research might benefit from exploring alternative categorization strategies, possibly incorporating a spectrum-based or a more granular classification system to capture the gradations of frailty more accurately.

However, it is important to acknowledge the limitations of this study, particularly its small sample size. This constraint makes it challenging to draw broad, generalizable conclusions. The findings, therefore, should be viewed as preliminary, serving primarily as a catalyst for further research. It is evident that additional studies with larger and more diverse populations are needed to deepen our understanding of frailty in aged-care settings. Such research will not only validate and potentially expand upon our findings but also contribute to the development of more effective assessment for frailty in these environments.

This study underscores the challenges in assessing frailty among aging populations, highlighting the imprecision and variability inherent in traditional tools. Our findings reveal the limitations of these tools in capturing the complex and multidimensional nature of frailty, suggesting that no single tool can encompass its entire spectrum. This complexity makes it difficult to endorse any specific frailty assessment tool for use in aged-care settings, pointing to a pressing need for more research to enhance these existing methodologies. In response to these challenges, the emerging field of geroscience offers a promising perspective by advocating for the integration of physiological biomarkers with traditional frailty assessments. This approach aims to improve the management of frailty in older individuals by incorporating key biomarkers such as mitochondrial dysfunction, proteostasis, stem cell dysfunction, and epigenetic changes, which have been shown to enhance early detection and intervention strategies (Lebrasseur et al., 2021). Researchers like Guerville et al. (2020) and Picca et al. (2020) support a multi-marker approach that merges these biomarkers with conventional frailty assessments to provide a more comprehensive evaluation of an individual's health status. While still in its early stages, this integrated approach could potentially offer new insights into how we manage and understand frailty in the elderly, suggesting that it is an area well-suited for further exploration and development.

Our findings contribute to the ongoing efforts aimed at refining frailty assessments. By highlighting key areas for further research, this study underscores the critical need for continuous improvement in assessment methods. We hope that our work will inspire further investigations, ultimately enhancing the effectiveness and accuracy of frailty assessments in clinical settings.

## Supplemental Material

Supplemental Material - Not All Frailty Assessments Are Created Equal: Comparability of Electronic Health Data-Based Frailty Assessments in Assessing Older People in Residential Care Supplemental Material for Not All Frailty Assessments Are Created Equal: Comparability of Electronic Health Data-Based Frailty Assessments in Assessing Older People in Residential Care by Jonathan Kong, Kelly Trinh, Kathrine Hammill, and Carla Chia-Ming Chen in Biological Research For Nursing
