# Supplementary material for: Not All Frailty Assessments Are Created Equal: Comparability of Electronic Health Data-Based Frailty Assessments in Assessing Older People in Residential Care
Source: Biol Res Nurs. 2024 May 13;26(4):526–36. doi: 10.1177/10998004241254459 (PMC11439236; doi:10.1177/10998004241254459)
Supplement: Supplemental Material - Not All Frailty Assessments Are Created Equal: Comparability of Electronic Health Data-Based Frailty Assessments in Assessing Older People in Residential Care [file sj-pdf-1-brn-10.1177_10998004241254459.pdf]

Appendix 1. The baseline characteristics of the residents based on the frailty groups for each frailty assessment instrument.

	**eFI**					**reFI**					**CFS**				
	Fit-Mildly Frail (n=176)	%	Moderately-Severely Frail (n=637)	%	p-value	Fit-Mildly Frail (n=328)	%	Moderately-Severely Frail (n=485)	%	p-value	Fit-Mildly Frail (n=64)	%	Moderately-Severely Frail (n=749)	%	p-value
Age (years)	84.1±8.01		87.1±7.07		<0.05	86.1±7.7		87.9±6.9		<0.05	86.3±6.8		86.4±7.4		0.928
Female	111	63.1	445	69.9	0.086	218	66.5	338	69.7	0.331	43	67.2	513	68.5	0.829
Male	65	36.9	192	30.1		110	33.5	147	30.3		21	32.8	236	31.5	
Top 20 Frailty Deficits															
Mobility Impairment	163	92.6	617	96.9	0.012	312	95.1	468	96.5	0.331	53	82.8	727	97.1	<0.05
Hypertension (HT)	78	44.3	494	77.6	<0.05						47	73.4	525	70.1	0.5739
Hypotension (HP)	4	2.3	31	4.9	<0.05						5	7.8	30	4.0	0.1498
HT or HP						182	55.5	404	83.3	<0.05					
Arthritis	83	47.2	440	69.1	<0.05	175	53.4	348	71.8	<0.05	35	54.7	488	65.2	0.093
Depression	92	52.3	406	63.7	<0.05	146	44.5	352	72.6	<0.05	31	48.4	467	62.3	<0.05
Dementia	89	50.6	350	54.9	0.302	170	51.8	269	55.5	0.308	24	37.5	415	55.4	<0.05
Pain	72	40.9	331	52.0	<0.05	116	35.4	287	59.2	<0.05	29	45.3	374	49.9	0.478
Heart Failure (Cardiac)	23	13.1	363	57.0	<0.05	108	32.9	297	61.2	<0.05	27	42.2	359	47.9	0.377
Visual Impairment	48	27.3	322	50.5	<0.05	116	35.4	254	52.4	<0.05	34	53.1	336	44.9	0.203
Incontinence	34	19.3	307	48.2	<0.05	102	31.1	239	49.3	<0.05	14	21.9	327	43.7	<0.05
Anxiety	63	35.8	263	41.3	0.188	86	26.2	240	49.5	<0.05	23	35.9	303	40.5	0.479
Polypharmacy	10	5.7	261	41.0	<0.05	44	13.4	227	46.8	<0.05	15	23.4	256	34.2	0.080
Cognition	50	28.4	209	32.8	0.267	81	24.7	178	36.7	<0.05	16	25.0	243	32.4	0.220
Osteoporosis	24	13.6	223	35.0	<0.05	63	19.2	184	37.9	<0.05	21	32.8	226	30.2	0.659
Cancer	54	30.7	192	30.1	0.890	76	23.2	170	35.1	<0.05	21	32.8	225	30.0	0.643
Respiratory System Disease	23	13.1	217	34.1	<0.05	57	17.4	183	37.7	<0.05	16	25.0	224	29.9	0.408
Falls	15	8.5	224	35.2	<0.05	57	17.4	182	37.5	<0.05	12	18.8	227	30.3	0.051
Fracture	20	11.4	191	30.0	<0.05	44	13.4	167	34.4	<0.05	16	25.0	195	26.0	0.856
Diabetes	19	10.8	181	28.4	<0.05	50	15.2	150	30.9	<0.05	13	20.3	187	25.0	0.406

	eFI					reFI					CFS				
	Fit-Mildly Frail (n=176)	%	Moderately-Severely Frail (n=637)	%	p-value	Fit-Mildly Frail (n=328)	%	Moderately-Severely Frail (n=485)	%	p-value	Fit-Mildly Frail (n=64)	%	Moderately-Severely Frail (n=749)	%	p-value
Hearing Loss	14	8.0	146	22.9	<0.05	44	13.4	116	23.9	<0.05	16	25.0	144	19.2	0.264
Chronic Kidney Disease	9	5.1	150	23.5	<0.05	34	10.4	125	25.8	<0.05	12	18.8	147	19.6	0.865

Values are presented as mean±standard deviation or number (%). Prevalence of frailty deficits and medical history (Top 20 list) were shown.

Appendix 2. Principal Component Analysis.

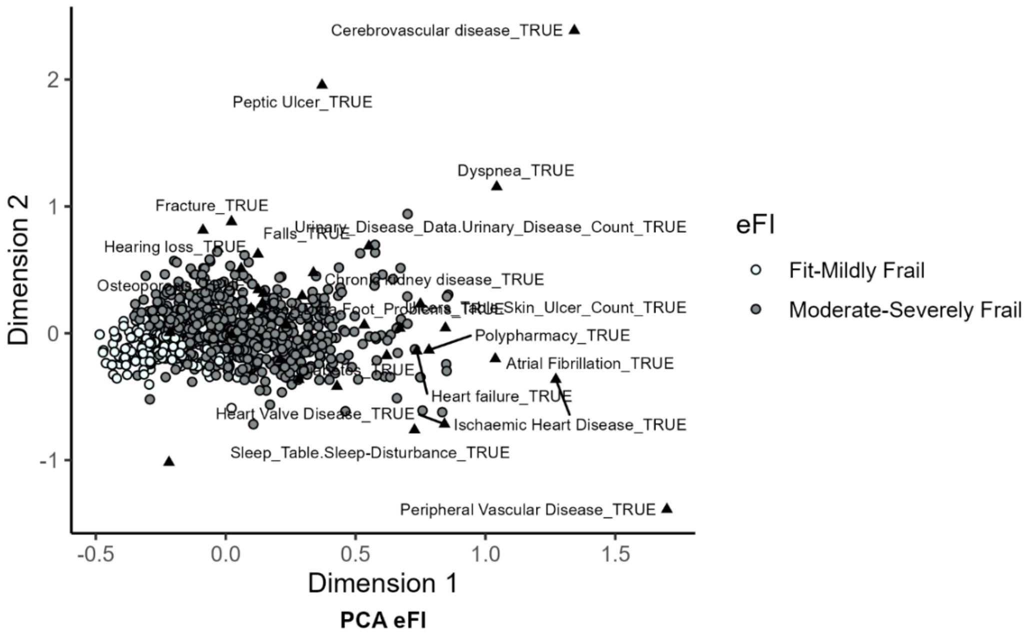

Supplementary Figure 1. PCA eFI.

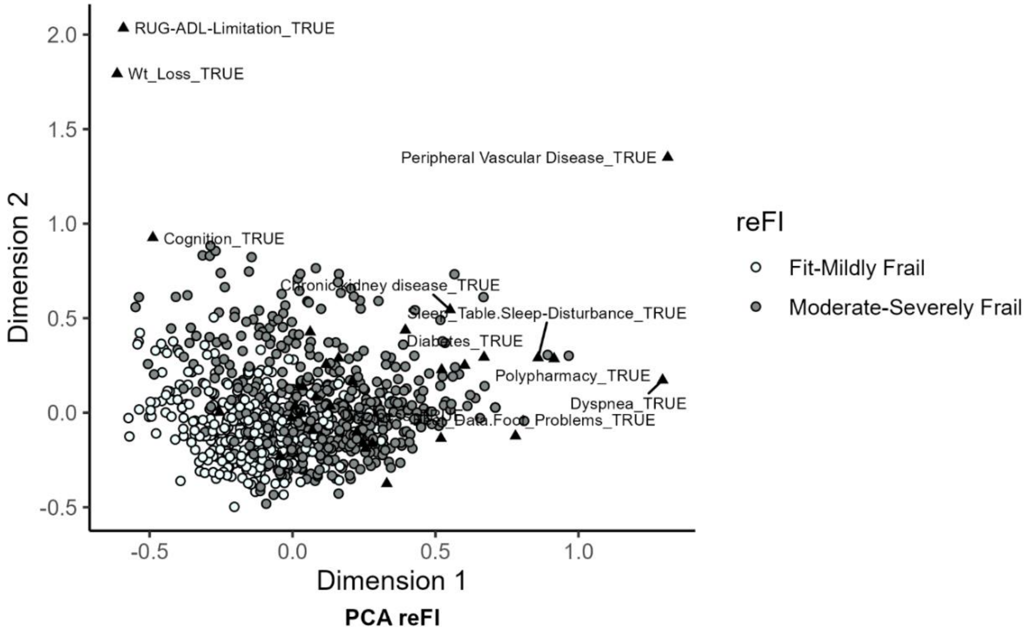

Supplementary Figure 2. PCA reFI.

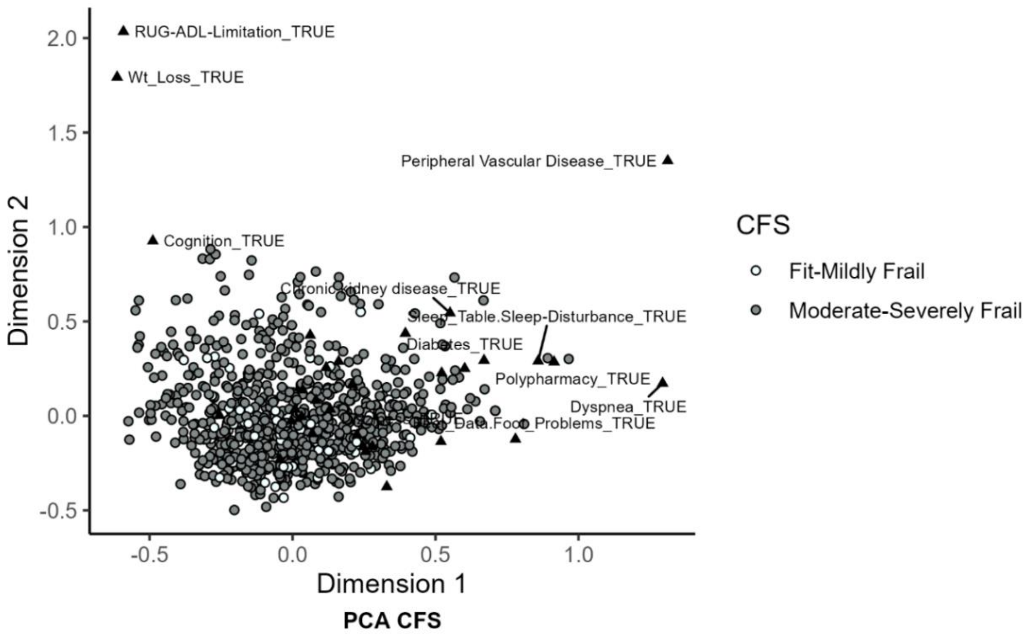

Supplementary Figure 3. PCA CFS.

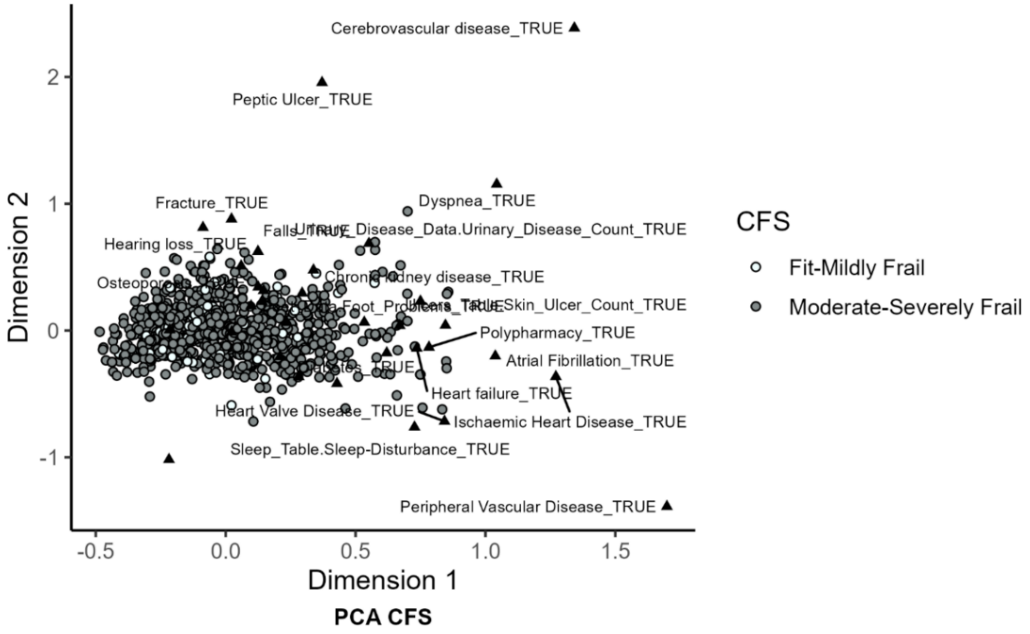

Supplementary Figure 4. PCA CFS
